# Removal of dichloromethane from waste gas streams using a hybrid bubble column/biofilter bioreactor

**DOI:** 10.1186/2052-336X-12-22

**Published:** 2014-01-09

**Authors:** Mehrnoosh Abtahi, Kazem Naddafi, Alireza Mesdaghinia, Kamyar Yaghmaeian, Ramin Nabizadeh, Nematollah Jaafarzadeh, Noushin Rastkari, Shahrokh Nazmara, Reza Saeedi

**Affiliations:** 1 Department of Environmental Health Engineering, School of Public Health and Institute for Environmental Research, Tehran University of Medical Sciences, P.O. Box 14155–6446, Tehran, Iran; 2 Environmental Technology Research Center, Ahvaz Jundishapur University of Medical Sciences, Ahvaz, Iran; 3 Center for Air Pollution Research, Institute for Environmental Research, Tehran University of Medical Sciences, Tehran, Iran; 4 Department of Public Health, Faculty of Health, Safety and Environment, Shahid Beheshti University of Medical Sciences, Tehran, Iran

**Keywords:** Dichloromethane, Waste gas streams, Bubble column bioreactor, Biofilter, HBCB bioreactor, Elimination capacity

## Abstract

The performance of a hybrid bubble column/biofilter (HBCB) bioreactor for the removal of dichloromethane (DCM) from waste gas streams was studied in continuous mode for several months. The HBCB bioreactor consisted of two compartments: bubble column bioreactor removing DCM from liquid phase and biofilter removing DCM from gas phase. Effect of inlet DCM concentration on the elimination capacity was examined in the DCM concentration range of 34–359 ppm with loading rates ranged from 2.2 to 22.8 g/m^3^.h and constant total empty bed retention time (EBRT) of 200 s. In the equal loading rates, the elimination capacity and removal efficiency of the biofilter were higher than the corresponding values of the bubble column bioreactor. The maximum elimination capacity of the HBCB bioreactor was determined to be 15.7 g/m^3^.h occurred in the highest loading rate of 22.8 g/m^3^.h with removal efficiency of 69%. The overall mineralization portion of the HBCB bioreactor was in the range of 72-79%. The mixed liquor acidic pH especially below 5.5 inhibited microbial activity and decreased the elimination capacity. Inhibitory effect of high ionic strength was initiated in the mixed liquor electrical conductivity of 12.2 mS/cm. This study indicated that the HBCB bioreactor could benefit from advantages of both bubble column and biofilter reactors and could remove DCM from waste gas streams in a better manner.

## Introduction

Dichloromethane (DCM, CH_2_Cl_2_), so-called methylene chloride, is an environmental contaminant of concern causing both acute and chronic health effects. This compound is a synthetic volatile organic compound (VOC) without known natural sources. DCM octanol-water partitioning coefficient (K_ow_) of 17.8 indicates that the chlorinated chemical is moderately hydrophobic and its large Henry’s Law constant of 0.0017 atm.m^3^/mol represents high volatility of the compound. DCM is used in many industrial processes such as paint stripping and removing, metal cleaning and degreasing, pharmaceutical manufacturing, adhesive manufacturing, polyurethane foam production, film base manufacturing, polycarbonate resin production and solvent formulation. Worldwide consumption of DCM has been estimated to be about 600,000 tonnes per year in 2004 [1-4].

Inhalation of ambient air is the principal route of human exposure to DCM, but a slight amount of DCM can be also absorbed by human body via drinking water and food. The health effects of acute (short-term) exposure to DCM through inhalation consist mainly of nervous system effects including decreased visual, auditory, and motor functions and the production of carboxyhaemoglobin (COHb), but these effects are reversible once exposure ceases. Long-term exposure to DCM has the potential to cause chronic health effects including central nervous system (CNS) damages, cardiac effects, liver and lung cancers and mammary gland tumors. The International Agency for Research on Cancer (IARC) classified DCM in Group 2B as possibly carcinogenic to humans. As a consequence of these adverse health effects, World Health Organization (WHO) has assigned an ambient air guideline value of 3 mg/m^3^ (0.866 parts per million, by volume (ppm)) for DCM [5-7].

There are several physico-chemical technologies such as thermal and catalytic incineration, adsorption and wet scrubbing for DCM removal from waste gas streams, but these methods require high capital investment and running costs especially when dealing with high gas flow rates containing low pollutant concentrations [8,9]. Biological processes as cheap, environmental friendly, simple and reliable technologies are promising alternatives to control DCM pollution of ambient air. Biological removal of DCM from waste gas streams was investigated in the several studies. Biodegradation of DCM produces hydrochloric acid (HCl), intermediate organic compounds, CO_2_, soluble microbial products and new microbial cells [8,10].

Bioreactors treating waste gases can be classified into two general categories; bioreactors removing pollutant in a liquid phase (such as continuously stirred tank and bubble column) and bioreactors removing pollutant in a gas phase (such as biofilter, biotrickling filter and bioscrubber). The best type of bioreactor is defined with regard to pollutant concentration, gas flow rate and physico-chemical prosperities of the pollutant and end products. Biofilter as a cost-effective reactor for degradation of poorly soluble VOCs is not appropriate for treatment of high concentrations of acid-producing pollutants such as DCM. On the other hand, bubble column bioreactor is not sensitive to high concentrations of acid-producing pollutants [11-14]. Therefore in this study, in order to benefit from advantages of both biofilter and bubble column bioreactor, a hybrid bubble column/biofilter (HBCB) bioreactor was developed and operated in continuous mode for several months to optimize removal of DCM from waste gas streams.

## Materials and methods

### Experimental set-up

**Figure 1:**
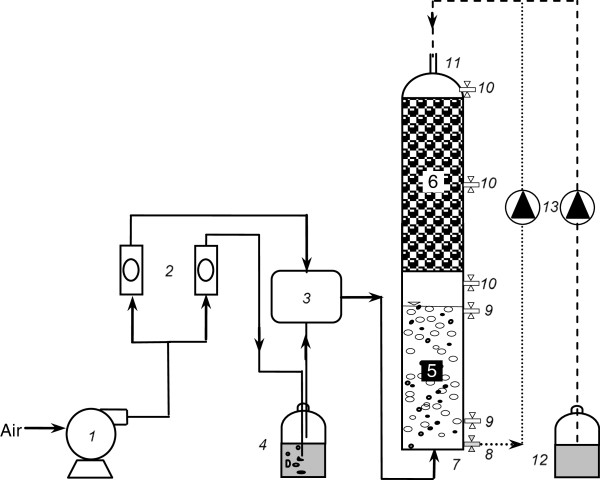
The experimental set-up used in this study: (1) air compressor, (2) air flowmeter, (3) mixing chamber, (4) DCM vaporization chamber, (5) bubble column bioreactor, (6) biofilter, (7) gas inlet, (8) drainage port, (9) liquor sampling port, (10) gas sampling port, (11) gas outlet, (12) nutrient reservoir and (13) peristaltic pumps.

The schematic diagram of the experimental set-up is shown in Figure 1. The experimental set composed of three parts: gas loading units, the HBCB bioreactor including the bubble column bioreactor and the biofilter and conditioning unit for humidification of the biofilter medium and nutrient and trace element supply. Polluted air (inlet gas) stream was prepared by mixing a large pure air stream with a small air containing DCM stream in a mixing chamber. The desirable concentrations of DCM in the inlet gas stream were obtained by adjusting the flow rates of the pure air and air containing DCM streams using the valves installed on the flowmeters. Air flow entrance to the bubble column bioreactor was conducted using an air diffuser. The inlet gas stream was passed through the reactor in an up flow mode. The HBCB bioreactor was constructed from a plexiglas tube with an inner diameter of 5 cm and effective bed heights of 25 and 38 cm for the bubble column bioreactor and biofilter, respectively. The volume of the bubble column bioreactor and biofilter were designed to be 500 and 750 mL, respectively. The biofilter was placed upon the bubble column bioreactor and was separated by a perforated plexiglas plate. This plate was kept biofilter media and redistributed air flow in the biofilter bed. The required humidity for microbial activity in the biofilter was mainly supplied by passing the influent air through the bubble column bioreactor. The growth media of the biofilter was polystyrene inert packing material (Bee-Cell 2000, DANAQ, Denmark) with bulk porosity of 87% and specific surface area of 650 m^2^/m^3^. A perforated plexiglas plate was placed above biofilter that facilitated distribution of nutrient and trace element solution.

### Addition of nutrients and trace elements

Nutrients and trace elements were added to the HBCB bioreactor in a regular manner to supply microbial growth requirements. Two stock solutions were prepared; nutrients and trace elements. The nutrient stock solution was prepared by use of deionized water and NH_4_Cl and NaH_2_PO_4_ at concentrations of 1911 and 387 mg/L, respectively. The ingredients of the trace element stock solution and their concentrations were FeSO_4_.7H_2_O at 500 mg/L, ZnSO_4_.7H_2_O at 400 mg/L, C_10_H_14_N_2_Na_2_O_8_.2H_2_O at 250 mg/L, CoCl_2_.6H_2_O at 50 mg/L, CuSO_4_.5H_2_O at 30 mg/L, MnCl_2_ at 20 mg/L, H_3_BO_3_ at 15 mg/L, NiCl_2_.6H_2_O at 10 mg/L and (NH_4_)_6_Mo_7_O_24_.4H_2_O at 10 mg/L. Addition of nutrients and trace elements to the HBCB bioreactor was performed once a day. To prepare nutrient and trace element solution for daily use, a volume of 1 mL from each stock solution was decanted into a volumetric flask and diluted to 100 mL by tap water. The prepared solution was poured from top of the biofilter and after passing through biofilter bed was added to mixed liquor of the bobble column bioreactor. After addition of the nutrient and trace element solution, in order to complete humidification of the biofilter medium and washout of excess biofilm and waste materials of DCM biodegradation, the mixed liquor was recirculated from top of the biofilter for 30 min. Finally, an equal volume of added solution to the mixed liquor was discharged from the bioreactor daily. With regard to the effective volume of the bubble column bioreactor (500 mL) and mixed liquor discharge regime (100 mL/d), the hydraulic retention time (HRT) in the bubble column bioreactor was 5 d.

### Microbial inoculation and start-up of bioreactor

The mixed microbial consortium was acquired from an activated sludge pilot plant treating 4-chlorophenol polluted wastewater. After microbial seeding, the HBCB bioreactor was run in mixed liquor recirculation mode (from bottom of the bobble column bioreactor to top of the biofilter) for 30 d to enrich DCM degrading mixed culture, acclimatize the bacteria by the substrate and accelerate biofilm development on the biofilter media. Following this period, the mixed liquor recirculation was stopped and normal operation of the HBCB bioreactor was started at a gradually increasing DCM concentration from 5 to 30 ppm during 30 d to complete start-up stage.

### Experimental procedure

All of the experiments were performed at constant gas flow rate of 0.375 L/min. The empty bed retention time (EBRT) of the bubble column bioreactor, biofilter and HBCB bioreactor were 80, 120 and 200 s. All of the experiments were conducted at ambient laboratory temperature (20?±?2°C).

Effect of inlet DCM concentration: The experiments were performed at five runs with variable inlet DCM concentrations from 34 to 359 ppm (34, 65, 121, 241 and 359 ppm) which was named as Run I, Run II, Run III, Run IV and Run V, respectively. Each experimental run was continued about one month to achieve steady-state condition. In order to investigate the performance of the HBCB bioreactor, DCM elimination capacity and efficiency, mineralization portion of DCM, intermediate organic compounds of DCM biodegradation, concentrations of the mixed liquor quality parameters including DCM, mixed liquor suspended solids (MLSS), chemical oxygen demand (COD), pH, electrical conductivity (EC) and chloride (Cl^-^) were determined in each experimental run. Because of HCl production during DCM biodegradation, pH of the mixed liquor decreased gradually, hence for continuity of microbial activity, pH of the mixed liquor was adjusted to about 8.0 by using NaOH on daily basis.

Effect of acidic pH: As mentioned above, pH of the bioreactor media decreased regularly and was adjusted to about 8.0. In order to investigate effect of acidic pH on the bioreactor performance, the HBCB bioreactor was operated at inlet DCM concentration of 240 ppm without pH adjustment for 21 d.

Effect of waste material accumulation: Effect of waste material accumulation on the HBCB bioreactor performance was studied at inlet DCM concentration of 240 ppm by removing the regular mixed liquor replacement by tap water (100 mL/d) for 42 d, therefore in this experiment HRT increased gradually.

### Analytical methods

DCM concentration in both gas and liquid samples was determined by a gas chromatograph (CP-3800, Varian) coupled with flame ionization detector (GC/FID). Type of the capillary column was CP-Sil 8 CB with length of 30 m, inner diameter of 0.32 mm and film thickness of 0.25 μm. The injection FID temperature was raised from 35°C (1 min) to 100°C at rate of 16°C/min and held for 5 min. Air samples of 200 μL were taken from the gas sampling ports placed on the experimental set-up and then injected to GC/FID with a 1 mL gas-tight syringe. Aqueous samples were taken of liquor sampling ports and then were analyzed by the headspace analytical technique. For this object, a sample volume of 5 mL was drawn with a 10 mL vial sealed with screw cap and polytetrafluoroethylene–silicon septum. Then, the aqueous samples were analyzed using the gas chromatographic system consisting of the GC/FID (the same instrument as above) equipped with a headspace (CombiPAL, CTC Analytics) [15,16]. To calculate mineralization portion of DCM, CO_2_ concentration was analyzed in gas samples using GC/FID same as DCM. Intermediate organic compounds of DCM biodegradation were also analyzed in both gas and mixed liquor samples using a gas chromatograph mass spectrometer (CP-3800, Varian/Saturn 2200). All of the other quality parameters of the mixed liquor including pH, EC, Cl^-^, MLSS and COD were measured according to the instructions of Standard Methods [15-17].

## Results and discussion

### Effect of inlet DCM concentration

**Figure 2:**
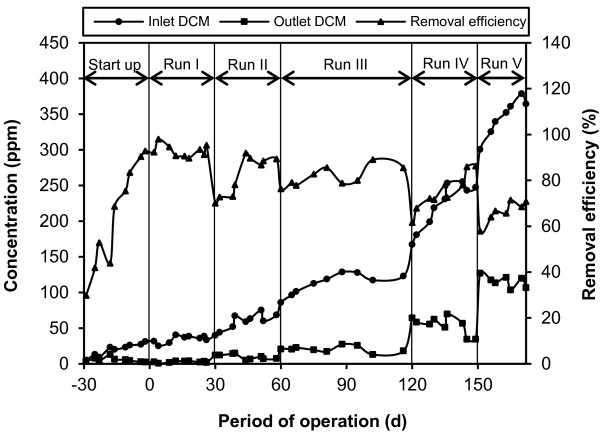
Profiles of inlet and outlet DCM concentrations and removal efficiency of the HBCB bioreactor during the experimental runs at different inlet DCM concentrations.

**Figure 3:**
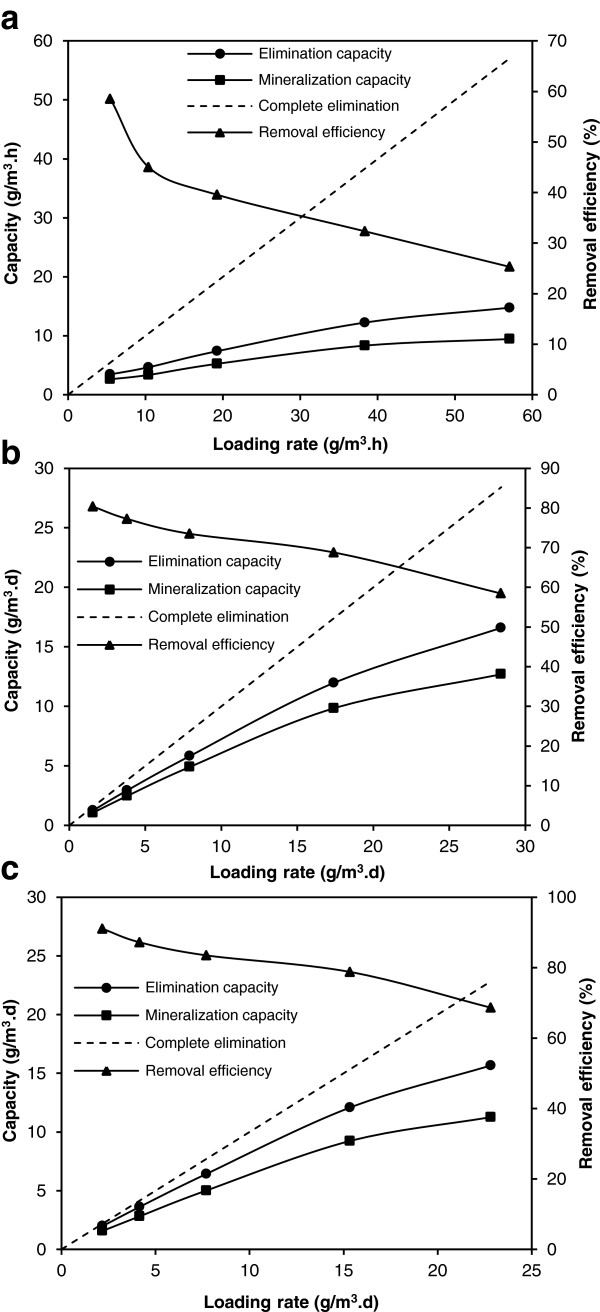
Effect of DCM loading rate on the elimination and mineralization capacities: (a) bubble column bioreactor, (b) biofilter and (c) HBCB bioreactor.

Figure 2 shows profiles of inlet and outlet DCM concentrations and removal efficiency of the HBCB bioreactor during the experimental runs. As indicated in Figure 2, by increasing inlet DCM concentration from 241 to 359 ppm, the removal efficiency decreased drastically. Effect of DCM loading rate on the elimination and mineralization capacities of the bubble column bioreactor, biofilter and HBCB bioreactor is presented in Figure 3. According to Figure 3, elimination capacity and removal efficiency of the biofilter were higher than the corresponding values of the bubble column bioreactor in the equal loading rates. The higher elimination capacity of bioreactors removing pollutant in a gas phase comparing with bioreactors removing pollutant in a liquid phase was also reported in the previous studies [8].

The maximum elimination capacity of the HBCB bioreactor was determined to be 15.7 g/m^3^.h occurred in the highest loading rate of 22.8 g/m^3^.h with removal efficiency of 69%. By increasing loading rate, the elimination and mineralization capacities increased and the removal efficiency and mineralization portion decreased in all of the bioreactors. The overall mineralization portion of the HBCB bioreactor was in the range of 72-79%. The mineralization portion of the bubble column bioreactor was also lower than that of the biofilter in the equal loading rate constantly. In the other hand, the calculation of the amount of produced biomass indicated that about 11% carbon of the removed DCM was converted to the biomass (data not shown). Therefore, the proportion of mineralization plus carbon absorbed to cell mass was in the range of 83-90% and the residue was appearing as the organic intermediate compounds. Lack of complete mineralization of DCM has also been reported in some other studies. Yu et al. [18] reported that in the DCM oxidation using UV/O_3_, the mineralization capacity was lower than the DCM elimination capacity in all of the experimental condition. According to De Best et al. [19], DCM mineralization in a packed bed bioreactor under the anoxic condition was not complete and formic acid (CH_2_O_2_) and acetic acid (C_2_H_4_O_2_) were observed as the intermediate compounds of DCM biodegradation.

In this study, we tried to identify the intermediate compounds in both the mixed liquor and gas stream. The detectable intermediates were the same in both the mixed liquor and gas stream and consisted of methanol (CH_4_O), formic acid, acetic acid, formaldehyde (CH_2_O) and carbonyl dichloride (CCl_2_O). Among them, the intermediate compounds of the both aerobic and anaerobic routes of DCM biodegradation were observed [20]. Since the concentration of dissolved oxygen in the mixed liquor was in the range of 6.8-8.7 mg/L which guaranteed aerobic conditions in bubble column bioreactor, anaerobic conditions might be occurred in depth of the biofilm in the biofilter due to the oxygen transfer limitation [21].

Due to the various experimental conditions employed in different studies, the maximum elimination capacity could not be solely a suitable measure for comparing different bioreactor and other parameters such as outlet concentration, removal efficiency, pH, temperature, etc. should also be taken into consideration. Ravi et al. [22] observed a maximum elimination capacity of 20.1 g/m^3^.h at an inlet loading rate of 31.5 g/m^3^.h (with removal efficiency of 64%) in a compost biofilter treating DCM vapors. Ergas et al. [23] investigated the removal of DCM in a biofilter and reported a maximum elimination capacity of 10.3 g/m^3^.h with 98% removal efficiency. With regard to outlet concentration and removal efficiency, the obtained maximum elimination capacity of the HBCB bioreactor for DCM was promising in comparison with that of the other bioreactors. In addition to suitable elimination capacity and high removal efficiency, other advantages of the HBCB bioreactor consisted of low wastewater generation, low liquid recirculation, regular humidification of the biofilter inlet gas by passing through the bubble column bioreactor, low pressure drop, no bed clogging, simultaneous removal of DCM from both liquid and gas phases, etc.; consequently, the HBCB bioreactor used in this study could be classified as an efficient bioreactor for DCM removal from waste gas streams.

**Figure 4:**
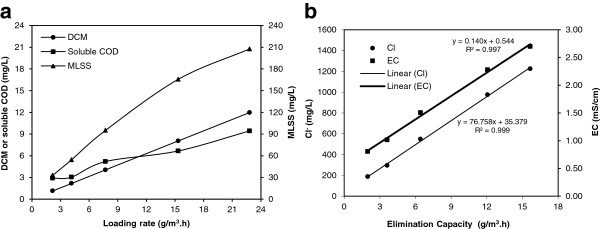
**Variations of the mixed liquor quality parameters at different inlet DCM concentrations: (a) DCM, soluble COD and MLSS versus loading rate and (b) Cl**^**- **^**and EC versus elimination capacity.**

Figure 4 shows variations of the mixed liquor quality parameters at different inlet DCM concentrations. As indicated in Figure 4, by increasing loading rate from 2.2 to 22.8 g/m^3^.h, DCM and soluble COD concentrations of the mixed liquor increased from 1.1 and 2.9 mg/L to 12.0 and 9.4 mg/L, respectively. Soluble COD composed of DCM, intermediate compounds of DCM microbial decomposition and soluble microbial products (SMPs) [24]. A nonlinear relationship was observed between MLSS (as an index for microbial quantity) and DCM loading rate, so that by increasing DCM loading rate from 2.2 to 22.8 g/m^3^.h, MLSS concentration of the mixed liquor increased from 33 and 208 mg/L.

Hydrochloric acid (HCl) was released into the mixed liquor during DCM biodegradation and NaCl was formed by neutralization of the acid using NaOH; therefore, linear relationships were found between elimination capacity and Cl^-^ and EC concentrations of the mixed liquor (Figure 4). According to linear regression analysis, Cl^-^ concentration and EC of the mixed liquor were increased 77 g/m^3^ and 140 μmoh/cm, respectively, per 1 g/m^3^.h elimination rate of DCM. The Cl^-^ generation was lower than the ones expected from the DCM elimination capacity and stoichiometric equation (2 moles Cl^-^ generation versus 1 mole DCM degradation). According to the stoichiometric equation, the expected increase in Cl^-^ concentration of mixed liquor per 1 g/m^3^.h elimination rate of DCM is 250 g/m^3^. Therefore, the observed value of increase in Cl^-^ concentration is about 31% of the stoichiometric amount. This observation can be attributed to incomplete mineralization (formation of chlorinated intermediates) and HCl fumigation. The highest Cl^-^ and EC concentrations were observed in Run V (with elimination capacity of 15.7 g/m^3^.h) to be 1225 mg/L and 2.7 mS/cm, respectively, these values were not inhibitor for microbial activity.

### Effect of acidic pH

**Figure 5:**
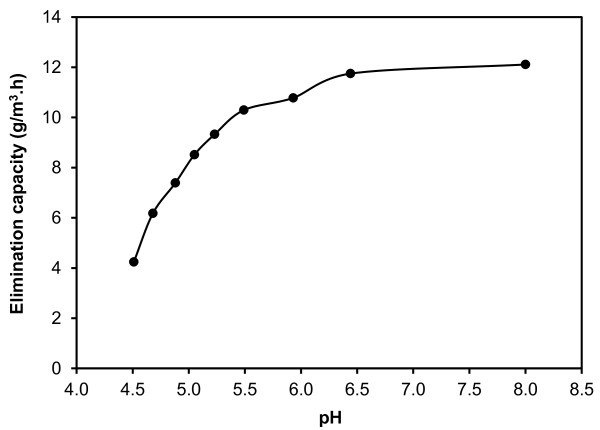
Effect of acidic pH on the elimination capacity of the HBCB bioreactor.

Effect of acidic pH on the performance of the HBCB bioreactor is depicted in Figure 5. According to Figure 5, effect of pH on the performance of the HBCB bioreactor started after 7 d by reduction of pH to 6.0 and increased during the experiment period (21 d) continuously. In pH values below 5.5, inhibitory effect of acidic pH on microbial activity increased drastically, so that by decreasing pH from 5.5 to 4.5, the elimination capacity decreased from 10.3 to 4.2 g/m^3^.h (about 65% decease in comparison with steady state operation). In order to prevent complete microbial inactivation, the experiment was not continued below pH value of 4.5 that occurred in 21st d. Although acidic pH decreased removal efficiency of DCM, but the HBCB bioreactor presented a promising resistance to pH reduction. This observation has significant practical importance as the HBCB would be flexible under actual operating conditions. Damaging effect of acidic pH on biodegradation of DCM was also reported by Jian-ming et al. [25]. Jianwei et al. [26] were examined the effect of acidic pH on the biofiltration performance of some pollutants from gas phase. The results showed that in the acidic pH (pH?=?4), the elimination rate of ethyl mercaptan and styrene were increased from 1.6 g/m^3^.h and 1.8 g/m^3^.h to 2.3 g/m^3^.h and 2.6 g/m^3^.h, respectively, whereas the elimination rate of butyric acid and ammonia were decreased from 2.1 g/m^3^.h and 1.9 g/m^3^.h to 1.7 g/m^3^.h and 1.3 g/m^3^.h, respectively. In the acidic pH, bacteria population was decreased and fungi and acidophilic thiobacteria became dominant in the bioreactor.

### Effect of waste material accumulation

**Figure 6:**
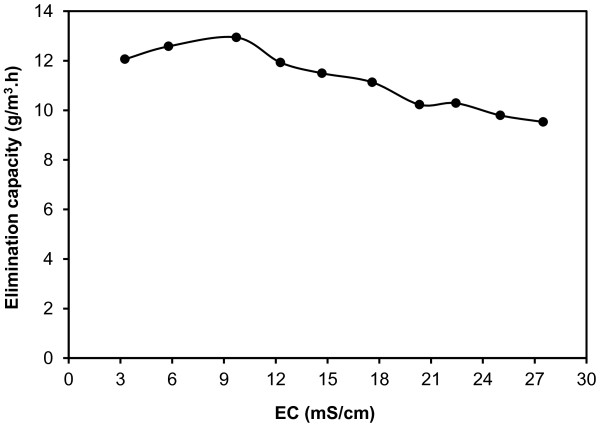
Effect of waste material accumulation on the elimination capacity of the HBCB bioreactor.

Figure 6 illustrates effect of waste material accumulation on the elimination capacity of the HBCB bioreactor. As indicated in Figure 6, in initial stage of the experiment (10 d), the elimination capacity of the HCBC bioreactor increased to 12.9 g/m^3^.h and then decreased with higher slope until the end of the experiment (42 d) to 9.5 g/m^3^.h that was about 80% of the elimination capacity in the regular operation. The inhibitory effect of the waste material accumulation on air treating bioreactors was also observed in the literature [25,27]. The tolerance limit of the HBCB bioreactor for EC was obtained 12.2 mS/cm. This value was lower than the EC tolerance limit of DCM biodegradation observed by Bailón et al. [8] to be 28 mS/cm. The initial increase of the elimination capacity could be as a result of the increased microbial mass (MLSS) in the mixed liquor. After 10 d, Cl^-^ (EC) concentration of the mixed liquor reached to the microbial inhibitory level and the effect of waste material accumulation became detectable and then increased until the end of the experiment gradually. These results indicated that in the HBCB bioreactor operation with inlet DCM concentration lower than 241 ppm, increasing HRT to 15 d would improve the bioreactor effectiveness and reduce wastewater generation.

## Conclusion

In this research, removal of DCM from waste gas streams using the HBCB bioreactor was investigated in EBRT of 200 s. The maximum elimination capacity of the HBCB bioreactor was determined to be 15.7 g/m^3^.h observed in the highest loading rate of 22.8 g/m^3^.h. The removal efficiency of the HBCB bioreactor was in the range of 69-91%. By increasing loading rate from 2.2 to 22.8 g/m^3^.d, the overall mineralization portion of the HBCB bioreactor decreased from 79 to 72%. The mixed liquor acidic pH especially below 5.5 and EC above 12.2 mS/cm inhibited microbial activity and decreased the elimination capacity. This study indicated that the HBCB bioreactor could be a feasible, flexible and efficient alternative for DCM removal from waste gas streams at full-scale.

## Abbreviations

CNS: Central nervous system; COD: Chemical oxygen demand; COHb: Carboxyhaemoglobin; DCM: Dichloromethane; EBRT: Empty bed retention time; EC: Electrical conductivity; GC/FID: Gas chromatograph/flame ionization detector; HBCB: Hybrid bubble column/biofilter; HRT: Hydraulic retention time; IARC: International agency for research on cancer; MLSS: Mixed liquor suspended solids; ppm: Parts per million, by volume; VOC: Volatile organic compound; WHO: World Health Organization.

## Competing interests

The authors declare that they have no competing interests.

## Authors’ contributions

MA participated in the design of the study, implemented the experiments, conducted data analysis and prepared the manuscript, KN and AM were the supervisors and participated in the design of the study, KY, RN, NJ and NR were the advisors and participated in analysis and interpretation of data, SN participated in the sample analysis and RS was involved in data analysis and drafting the manuscript. All authors read and approved the final manuscript.
